# Baseline ^18^F-FDG PET/CT radiomics for prognosis prediction in diffuse large B cell lymphoma

**DOI:** 10.1186/s13550-023-01047-5

**Published:** 2023-10-26

**Authors:** Fenglian Jing, Yunuan Liu, Xinming Zhao, Na Wang, Meng Dai, Xiaolin Chen, Zhaoqi Zhang, Jingmian Zhang, Jianfang Wang, Yingchen Wang

**Affiliations:** 1 Department of Nuclear Medicine, The Fourth Hospital of Hebei Medical University, 12 Jiankang Road, Shijiazhuang, 050011 Hebei China; 2 Hebei Provincial Key Laboratory of Tumor Microenvironment and Drug Resistance, Shijiazhuang, 050011 Hebei China

**Keywords:** FDG PET/CT, Diffuse large B-cell lymphoma, Risk stratification, Prognosis, Machine learning, Radiomics

## Abstract

**Background:**

Diffuse large B-cell lymphoma (DLBCL) is the most common subtype of non-Hodgkin lymphoma in adults. Standard treatment includes chemoimmunotherapy with R-CHOP or similar regimens. Despite treatment advancements, many patients with DLBCL experience refractory disease or relapse. While baseline ^18^F-fluorodeoxyglucose positron emission tomography (^18^F-FDG PET) parameters have shown promise in predicting survival, they may not fully capture lesion heterogeneity. This study aimed to assess the prognostic value of baseline ^18^F-FDG PET radiomics features in comparison with clinical factors and metabolic parameters for assessing 2-year progression-free survival (PFS) and 5-year overall survival (OS) in patients with DLBCL.

**Results:**

A total of 201 patients with DLBCL were enrolled in this study, and 1328 radiomics features were extracted. The radiomics signatures, clinical factors, and metabolic parameters showed significant prognostic value for individualized prognosis prediction in patients with DLBCL. Radiomics signatures showed the lowest Akaike information criterion (AIC) value and highest Harrell’s concordance index (C-index) value in comparison with clinical factors and metabolic parameters for both PFS (AIC: 571.688 vs. 596.040 vs. 576.481; C-index: 0.732 vs. 0.658 vs. 0.702, respectively) and OS (AIC: 339.843 vs. 363.671 vs. 358.412; C-index: 0.759 vs. 0.667 vs. 0.659, respectively). Statistically significant differences were observed in the area under the curve (AUC) values between the radiomics signatures and clinical factors for both PFS (AUC: 0.768 vs. 0.681, *P* = 0.017) and OS (AUC: 0.767 vs. 0.667, *P* = 0.023). For OS, the AUC of the radiomics signatures were significantly higher than those of metabolic parameters (AUC: 0.767 vs. 0.688, *P* = 0.007). However, for PFS, no significant difference was observed between the radiomics signatures and metabolic parameters (AUC: 0.768 vs. 0.756, *P* = 0.654). The combined model and the best-performing individual model (radiomics signatures) alone showed no significant difference for both PFS (AUC: 0.784 vs. 0.768, *P* = 0.163) or OS (AUC: 0.772 vs. 0.767, *P* = 0.403).

**Conclusions:**

Radiomics signatures derived from PET images showed the high predictive power for progression in patients with DLBCL. The combination of radiomics signatures, clinical factors, and metabolic parameters may not significantly improve predictive value beyond that of radiomics signatures alone.

**Supplementary Information:**

The online version contains supplementary material available at 10.1186/s13550-023-01047-5.

## Background

Diffuse large B-cell lymphoma (DLBCL) is the most common subtype of non-Hodgkin lymphoma (NHL) in adults [1]. The rituximab, cyclophosphamide, doxorubicin, vincristine, and prednisone (R-CHOP) or R-CHOP-like chemoimmunotherapy regimens are the standard treatment regimens for DLBCL [2]. The International Prognostic Index (IPI), a clinically based parameter, has been widely used for over 30 years to categorize patients with DLBCL into low- and high-risk groups on the basis of age, Ann Arbor stage, performance status, number of extranodal sites, and serum lactate dehydrogenase (LDH) level [3]. The enhanced National Comprehensive Cancer Network-IPI (NCCN-IPI) has further improved prognostic accuracy by re-evaluating age and LDH classifications and considering the involvement of specific extranodal sites [4]. β2-microglobulin (β2-MG), a protein synthesized in all nucleated cells and a component of the major histocompatibility complex class I antigen, has also been identified as a significant prognostic factor for patients with DLBCL [5, 6]. Despite advancements in treatment, approximately 40% of patients with DLBCL still experience primary refractory disease or relapse [7].

In recent years, significant advancements have been made in the utilization of baseline ^18^F-fluorodeoxyglucose positron emission tomography (^18^F-FDG PET) parameters to assess patients with DLBCL. Several parameters that reflect the total tumor burden and its dissemination, especially total metabolic tumor volume (TMTV) and the largest distance between two lesions (Dmax), have been proven to be robust indicators of disease progression-free survival (PFS) and overall survival (OS) in patients with DLBCL [8–13]. However, these PET parameters alone do not fully capture the metabolic heterogeneity and shape characteristics of the lesions, which have been independently identified as important prognostic factors for patients with DLBCL [14]. Fortunately, with the emergence of artificial intelligence, a novel approach known as radiomics has shown promise in extracting implicit features from PET images, thereby providing valuable prognostic information [15]. Several studies have already delved into the potential of baseline ^18^F-FDG PET radiomics in predicting outcomes for DLBCL patients [16–19].

This study aimed to develop and evaluate the prognostic significance of baseline radiomics signatures in comparison with clinical factors and conventional PET parameters in patients with DLBCL.

## Methods

### Study cohort

This retrospective study was conducted at the Fourth Hospital of Hebei Medical University (HBMU), and ethical approval was obtained from the institutional review board. The study was conducted between March 2012 and December 2021 and included patients diagnosed with new-onset diffuse large B-cell lymphoma (DLBCL). The inclusion criteria were as follows: (1) histologically confirmed DLBCL, (2) age 18 years or older, (3) prior ^18^F-FDG PET/CT imaging before treatment, and (4) initiation of treatment with R-CHOP or R-CHOP-like regimens. Patients with incomplete clinical data, concurrent central nervous system lymphomas, or other malignancies were excluded from the study. The institutional review board waived the requirement for obtaining written informed consent from the patients.

**Fig. 1:**
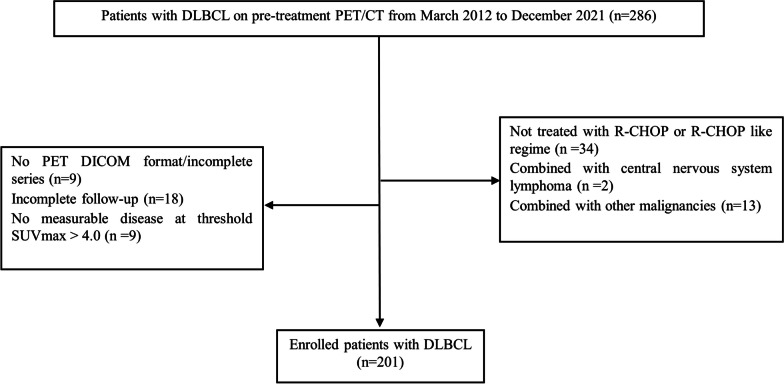
Flowchart of DLBCL patient selection

Figure 1 presents a flowchart illustrating the patient-selection process. A total of 201 patients diagnosed with DLBCL (112 men and 89 women) were included in the cohort. The average age of the patients was 54.80 ± 15.00 years (range, 18 to 84 years). Various clinical characteristics were recorded, including age, sex, serum lactate dehydrogenase (LDH) level, serum β2-MG level, Ann Arbor stage, performance status, involvement of important extranodal organs, B symptoms, and pathological type. The NCCN-IPI was calculated using a previously established method [3].

Follow-up assessments were conducted after treatment completion and continued until December 2022, with a minimum follow-up duration of 12 months or until death. This study assessed the prognosis of patients with DLBCL by using 2-year PFS and 5-year OS as the primary endpoints. PFS was defined as the period from initial diagnosis to the occurrence of disease relapse, progression, or death from any cause. Similarly, the OS was measured from the time of diagnosis to death from any cause.

### PET/CT acquisition and reconstruction

^18^F-FDG PET/CT scans were conducted using PHILIPS GEMINI GXL16 and PHILIPS Vereos scanners. Before the scans, patients had a fasting period of at least 6 h, and their blood glucose levels were required to be < 11.1 mmol/L. The PET/CT scans covered the entire body from the base of the skull to the upper thigh. The scans were performed approximately 60 ± 5 min after intravenous injection of 3.7–5.55 MBq/kg ^18^F-FDG. CT acquisition data were used to correct for attenuation. The details of PET/CT image acquisition parameters are displayed in Additional file 1: Table s1.

### PET image segmentation and radiomics feature extraction

The volumes of interest (VOIs) in the PET images were semi-automatically delineated using the LIFEx software (version 7.3.0; https://www.lifexsoft.org/) [20]. Two experienced nuclear medicine physicians who were blinded to the outcomes performed the VOI segmentation using a fixed threshold of SUV ≥ 4.0 [21]. Non-tumor FDG-avid regions (eg, obvious physiological uptake, kidney, ureter, bladder) were deleted, and non-tumor regions adjacent to tumor regions were manually removed. For each patient, conventional PET metabolic parameters of patient-level lesions, such as SUVmax, Dmax, and TMTV, were calculated using LIFEx. In cases with only one detectable lesion, the Dmax was recorded as 0 cm. To account for variations in patient size and height, Dmax was normalized to the patient’s body surface area (BSA) to derive the standardized Dmax (SDmax). The BSA was calculated as follows: $\sqrt {({\text{weight}} \times {\text{ height}}) / 3600}$. TMTV was extracted using the “Union of all visible ROI” function.

In total, 1328 radiomics features based on TMTV were extracted using open-source PyRadiomics (http://www.radiomics.io/pyradiomics.html) [22–24]. These features encompassed first-order statistical features, texture features extracted from the original or filtered images, and shape descriptors. The extracted radiomics features are displayed in Additional file 2: Table s2. All radiomics features were standardized using the z-score standardization method to reduce the influence of differences in size, characteristics, and distribution.

### Feature selection and model construction

The least absolute shrinkage and selection operator (LASSO) Cox regression algorithm, which utilizes tenfold cross-validation, was employed to select influential radiomics signatures with nonzero coefficients to build the radiomics model [25]. The radiomics score (rad-score) was computed by summing the selected features, with each feature being weighted by its corresponding LASSO coefficient.

NCCN-IPI and the β2-MG level were included as clinical predictors. Among metabolic parameters, SUVmax, SDmax, and TMTV were chosen. The optimal cutoff values of SUVmax, SDmax, and TMTV for PFS and OS were determined by maximizing the Youden index of the receiver operating characteristic (ROC) curve. Subsequently, univariate and multivariate Cox proportional-hazard regressions were conducted to select independent variables for constructing the clinical- and metabolic parameters- models. The clinical score (c-score) and metabolic parameters score (m-score) were derived by aggregating the chosen independent variables, where each variable was weighted according to its respective COX coefficient.

These scores were used to classify patients into low- and high-risk groups, with the best cutoff values determined using the ROC curve.

### Model evaluation and validation

The fitness of the three models was evaluated using the Akaike information criterion (AIC) [26]. A smaller AIC value indicated a better fit for the model. Generally, AIC value differences ≥ 10 indicate a significant improvement in model fitness, differences > 2 but < 10 demonstrate an improved fit, and differences < 2 between models indicate no significant improvement in model fitness [27]. The Harrell’s concordance index (C-index), which corresponds to the area under the ROC curve, was used to measure the predictive capability of the models. A C-index of 0.5 represents a random guess; a C-index of 0.7 indicates acceptable discrimination; and a C-index of 1.0 signifies perfect discrimination. For internal verification, a bootstrap resampling method (B = 1000) was employed to obtain the corrected C-index of the models [28].

The differences in the area under the curve (AUC) values in the three models were compared using the DeLong nonparametric test (MedCalc19.6.4) [29]. This test allows for a statistical comparison of the AUCs and provides insights into the performance differences between the models. Subsequently, a combined model incorporating the three individual models was developed and compared to the model with the best prediction performance. This comparison aimed to assess whether the combination of models improved the predictive capability.

### Statistical analysis

Statistical analyses were performed using SPSS software (version 26.0, IBM) and R statistical software (version 4.2.2) (http://www.R-project.org). The associations of the rad-score, c-score, and m-score with the type of scanner used (PHILIPS GEMINI GXL16 or PHILIPS Vereos) were compared using the Mann–Whitney U test. Kaplan–Meier analysis and log-rank tests were used to assess and compare the survival outcomes among the three models. A *P*-value of less than 0.05 was considered statistically significant.

## Results

### Patient baseline characteristics

The median PFS was 22.5 months, and the median OS was 28.5 months. During the follow-up period, 70 patients experienced disease relapse or progression and 39 died. The 2-year PFS rate was 70.1% (141/201), and the 5-year OS rate was 81.1% (163/201). Table 1 summarizes the clinical characteristics of the enrolled patients.

**Table 1:** Clinical characteristics of enrolled patients

Patient characteristics	Overall (n)	Percentage (%)
Age (years)		
≤ 40	41	20.4
41–60	72	35.8
61–75	78	38.8
> 75	10	5.0
Gender		
Male	112	55.7
Female	89	44.3
LDH ratio		
≤ 1	118	58.7
2–3	74	36.8
> 3	9	4.5
β2-MG ratio		
≤ 1	138	68.7
> 1	63	31.3
Ann Arbor stage		
I–II	77	38.3
III–IV	124	61.7
ECOG PS		
< 2	145	72.1
≥ 2	56	27.9
Involvement of extranodal important organs		
Yes	86	42.8
No	115	57.2
B symptoms		
Yes	69	34.3
No	132	65.7
Pathological type		
GCB	77	38.3
Non-GCB	124	61.7
PET/CT scanner		
PHILIPS GEMINI GXL16	93	46.3
PHILIPS Vereos	108	53.7

LDH lactate dehydrogenase, β2-MG β2-microglobulin, ECOG PS Eastern Cooperative Oncology Group performance status, GCB germinal center B-cell like.

### Feature analysis and model establishment

**Fig. 2:**
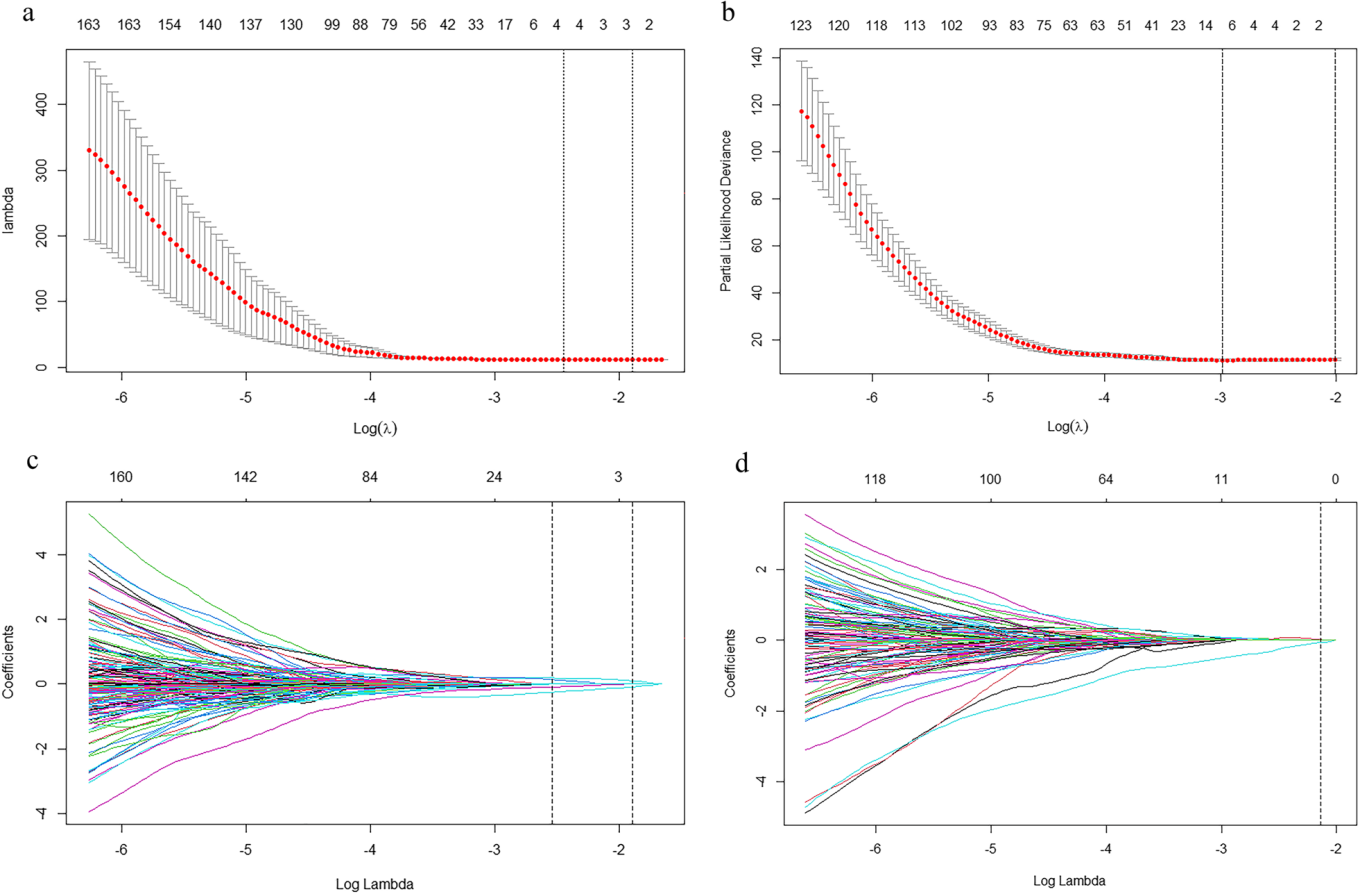
The least absolute shrinkage and selection operator (LASSO) Cox regression algorithm were used to select optimal radiomic features. The selected tuning parameter (Lambda) in LASSO model through tenfold cross-validation were with minimum criteria in predicting (**a**) PFS and (**b**) OS. LASSO coefficient distributions of the radiomic features in predicting (**c**) PFS and (**d**) OS. The dotted vertical lines indicate the optimal values using the minimum criteria

Figure 2 illustrates the results of the LASSO-Cox analysis, where among the 1328 radiomics features extracted based on TMTV, 4 features with nonzero coefficients were identified for predicting the 2-year PFS, and 10 additional features were identified for predicting the 5-year OS. The selected features and their corresponding coefficients are listed in Table 2. Radiomics signatures were constructed on the basis of these features. The rad-score was calculated for each patient to predict PFS and OS. The optimal cutoff values for the rad-score were determined to be 0.054 for PFS and 0.114 for OS.

**Table 2:** Results of radiomic feature selection for PFS and OS

End points	Feature name	Coefficient
PFS	original_shape_Maximum2DDiameterSlice	0.184
	squareroot_glrlm_LongRunLowGrayLevelEmphasis	0.107
	wavelet-LHH_glszm_SizeZoneNonUniformityNormalized	− 0.216
	wavelet-HHH_glszm_SizeZoneNonUniformityNormalized	− 0.070
OS	original_shape_Maximum2DDiameterSlice	0.034
	original_firstorder_Skewness	− 0.061
	original_glszm_SizeZoneNonUniformity	0.077
	exponential_glszm_SizeZoneNonUniformity	0.050
	square_firstorder_Median	0.019
	square_firstorder_Minimum	0.006
	wavelet-LLH_glszm_LowGrayLevelZoneEmphasis	0.030
	wavelet-HHL_glszm_SizeZoneNonUniformity	0.075
	wavelet-HHH_glszm_SizeZoneNonUniformityNormalized	− 0.507
	wavelet-HHH_glszm_SmallAreaLowGrayLevelEmphasis	− 0.018

The NCCN-IPI was considered as a multiple categorical variable in the analysis. The median β2-MG level was 2.22 mg/L (range, 0.61–9.90 mg/L). The β2-MG levels were categorized on the basis of the upper limit of the normal range. Univariate Cox regression analysis revealed that both the NCCN-IPI and β2-MG level were significantly associated with PFS and OS. Subsequently, multivariate analysis showed that the NCCN-IPI and β2-MG level were independent predictors of PFS. However, only the NCCN-IPI was identified as an independent predictor of OS. Please refer to Table 3 for the detailed results. The c-scores were then calculated to predict PFS and OS, with the most discriminative cutoff values of 1.197 and 0.875, respectively.

**Table 3:** Clinical predictors and metabolic parameters of Cox regression analysis for predicting PFS and OS

Category	End points	Variables	Univariate analysis			Multivariate analysis		
			HR	95%CI	*P*	HR	95%CI	*P*
Clinical predictors	PFS	NCCN-IPI	1.331	1.145–1.548	< 0.001	1.215	1.021–1.445	0.028
		β2-MG	2.668	1.606–4.433	< 0.001	1.904	1.060–3.421	0.031
	OS	NCCN-IPI	1.419	1.174–1.715	< 0.001	1.294	1.041–1.607	0.020
		β2-MG	3.015	1.589–5.719	< 0.001	1.933	0.927–4.030	0.079
Metabolic parameters	PFS	SUVmax	1.352	0.814–2.247	0.244	–	–	*–*
		SDmax	5.592	2.652–11.790	< 0.001	4.758	2.236–10.120	< 0.001
		TMTV	2.816	1.654–4.796	< 0.001	2.186	1.275–3.750	0.004
	OS	SUVmax	1.664	0.849–3.262	0.138	–	–	–
		SDmax	4.499	2.126–9.517	< 0.001	3.939	1.832–8.469	< 0.001
		TMTV	2.441	1.209–4.932	0.013	1.797	0.874–3.697	0.111

PFS progression-free survival, OS overall survival, NCCN-IPI National Comprehensive Cancer Network-International Prognostic Index, β2-MG β2-microglobulin, SUVmax maximum standardized uptake value, Dmax the largest distance between two lesions, SDmax standardized Dmax, TMTV total metabolic tumor volume, HR hazard ratio, CI confidence interval.

The study results revealed that the median SUVmax was 19.98 (range, 2.57–58.43). To classify patients into low or high SUVmax groups, the optimal cutoff values of PFS and OS were 20.48 and 26.24, respectively. The median SDmax was 5.92 cm^−1^ (range, 0–62.59 cm^−1^). For stratifying patients into low or high SDmax groups, the optimal cutoff values of PFS and OS were 2.96 and 7.83 cm^−1^, respectively. Median baseline TMTV was 120.13 mL (range, 1.86–2244.16 mL). Using ROC cutoff values of 166.78 and 102.72 mL for PFS and OS, respectively, the patients were classified into low- or high-TMTV groups. Univariate Cox analysis showed significant associations between SDmax and TMTV and both PFS and OS, whereas SUVmax was not significantly associated with either PFS or OS. In the multivariate Cox analysis, SDmax and TMTV were identified as independent predictors of PFS, whereas only SDmax remained an independent predictor of OS. Further details are provided in Table 3. The m-scores were calculated to predict PFS and OS with optimal cutoff values of 1.951 and 0.686, respectively.

### Model performance assessment and validation

**Fig. 3:**
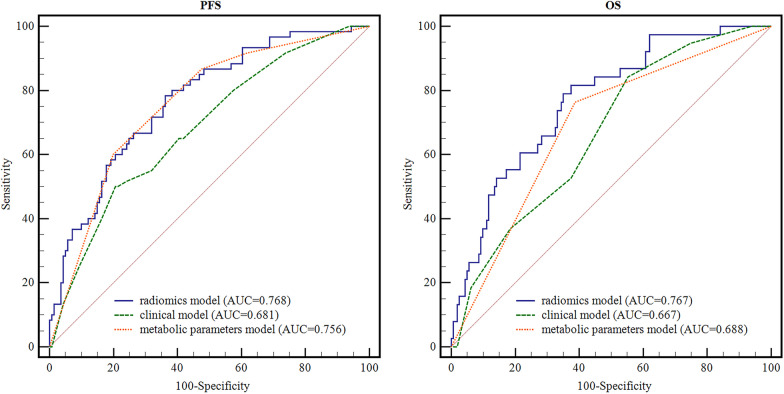
Time-dependent ROC curves of radiomics-, clinical-, and metabolic parameters model in predicting PFS and OS

For the 2-year PFS prediction, the radiomics signatures exhibited a lower AIC value (571.688) than clinical factors (596.040) and metabolic parameters (576.481). All differences of > 2 in AIC values among the three models demonstrated an improvement in model fitness. The C-indices of the radiomics signatures, clinical factors, and metabolic parameters were 0.732, 0.658, and 0.702, respectively. The C-indices of the models corrected using bootstrap resampling were 0.730, 0.655, and 0.702, respectively (Table 4). Statistically significant differences were observed in the AUCs between the radiomics signatures and clinical factors (AUC: 0.768 vs. 0.681, *P* = 0.017). However, no significant difference was observed between the radiomics signatures and metabolic parameters (AUC: 0.768 vs. 0.756, *P* = 0.654). Additionally, no significant differences were observed between the clinical factors and metabolic parameters (AUC: 0.681 vs. 0.756, *P* = 0.054; Fig. 3).

**Table 4:** The results of AIC value, Harrell’s C-index and corrected C-index for PFS and OS

	AIC	C-index (95%CI)	corrected C-index (95%CI)
PFS			
Radiomics signatures	571.688	0.732 (0.700–0.764)	0.730 (0.668–0.798)
Clinical factors	596.040	0.658 (0.624–0.693)	0.655 (0.588–0.725)
Metabolic parameters	576.481	0.702 (0.670–0.735)	0.702 (0.642–0.769)
OS			
Radiomics signatures	339.843	0.759 (0.716–0.803)	0.773 (0.692–0.857)
Clinical factors	363.671	0.667 (0.625–0.708)	0.665 (0.585–0.744)
Metabolic parameters	358.412	0.659 (0.620–0.698)	0.660 (0.585–0.740)

For the 5-year OS, the AIC value of the radiomics signatures was 339.843, which was also the lowest among the three models. The AIC values for the clinical factor and metabolic parameters models were 363.671 and 358.412, respectively. All differences > 10 in AIC values indicated a significant improvement in model fitting. The C-indices of the radiomics signatures, clinical factors, and metabolic parameters were 0.759, 0.667, and 0.659, respectively. The corrected C-indices of the models were 0.773, 0.665, and 0.660, respectively (Table 4). The AUCs of the radiomics signatures were significantly higher than those of both clinical factors (AUC: 0.767 vs. 0.667, *P* = 0.023) and metabolic parameters (AUC: 0.767 vs. 0.688, *P* = 0.007), although there were no significant differences between clinical factors and metabolic parameters (AUC: 0.667 vs. 0.688, *P* = 0.621; Fig. 3).

The AIC value of the combined model was 567.066 for PFS, and differences of > 2 but < 10 between the combined model and radiomic signatures demonstrated an improved fit. Meanwhile, the AIC value of the combined model was 339.546 for OS, and differences of < 2 between the combined model and radiomic signatures revealed no improvement in model fitting. The C-index values of the combined models were 0.739 (95% CI: 0.707–0.771) for PFS and 0.763 (95% CI: 0.721–0.805) for OS. No significant differences were observed between the combined model and radiomics signatures for PFS (*P* = 0.163) or OS (*P* = 0.403).

### Scanner comparability

No statistically significant differences were observed among the rad-score, c-score, and m-score in relation to the type of scanner used (PHILIPS GEMINI GXL16 or PHILIPS Vereos) for PFS (*P* = 0.284, 0.102, and 0.503, respectively) and OS (*P* = 0.693, 0.087, and 0.686, respectively).

### Survival prediction and risk stratification

**Fig. 4:**
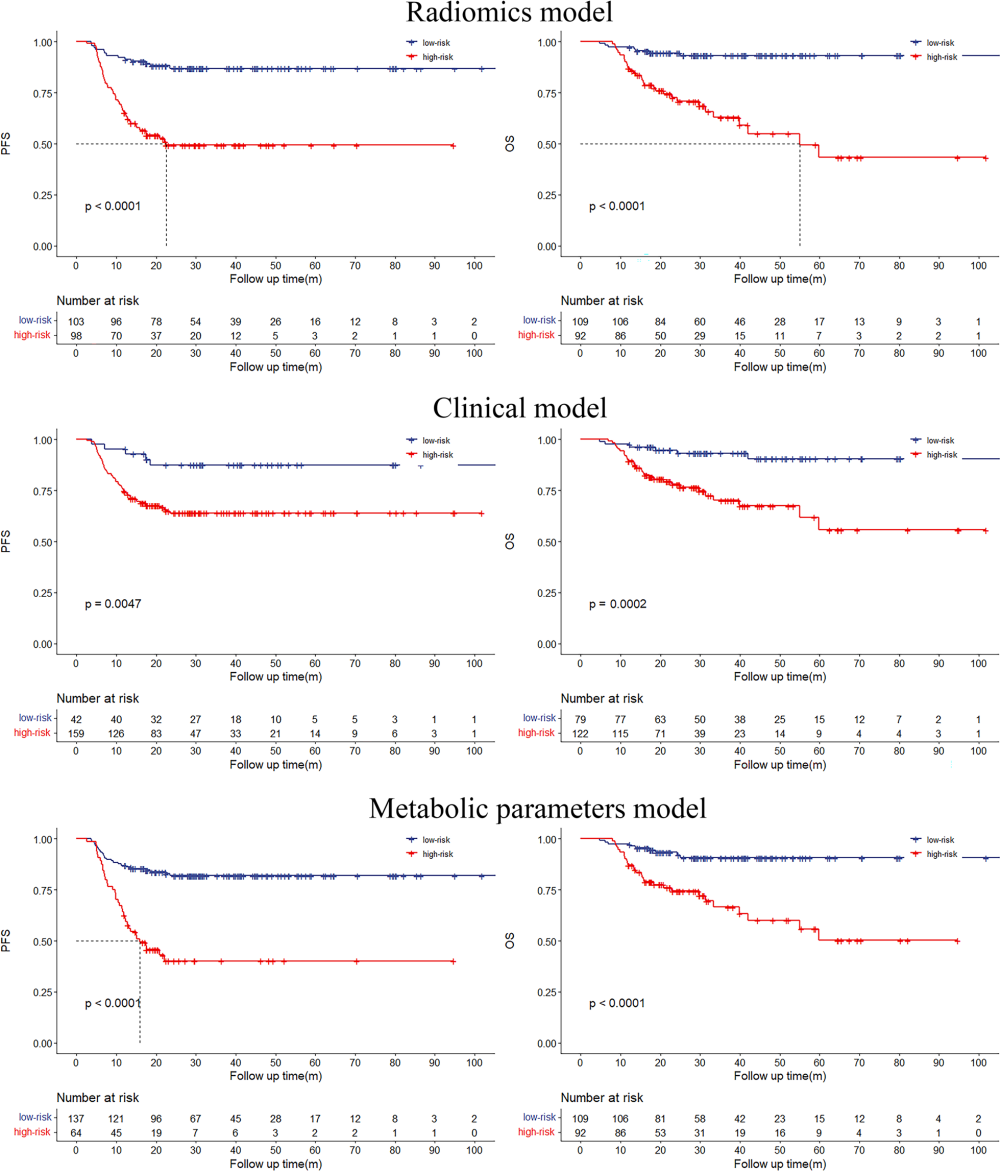
Kaplan–Meier estimates of PFS and OS in models and log-rank *P* value are reported

The Kaplan–Meier curves indicated that the recurrence and death rates of patients in the high-risk group were significantly higher than those in the low-risk group in all three models (all *P* < 0.05) for PFS and OS (Fig. 4). For PFS, the univariate hazard ratios (HRs) for radiomic signatures (8.546) were higher than those for clinical factors (2.712) and metabolic parameters (2.718). The radiomics score below 0.054 indicates a low risk of recurrence, while the score above 0.054 indicates a higher risk of recurrence. For OS, univariate HRs for radiomics signatures were also the highest for the three models (8.367) in comparison with the clinical factors model (2.718) and the metabolic parameters model (2.995; Table 5). Similarly, the score below 0.114 suggests a low risk of death, whereas the score above 0.114 indicates a higher risk of death.

**Table 5:** The results of HRs and log-rank P value for PFS and OS

	HR	HR (95%CI)	*P*
PFS			
Radiomics signatures	8.546	4.355–16.770	< 0.001
Clinical factors	2.712	1.725–4.264	< 0.001
Metabolic parameters	2.718	1.882–3.926	< 0.001
OS			
Radiomics signatures	8.367	3.947–17.740	< 0.001
Clinical factors	2.718	1.583- 4.669	< 0.001
Metabolic parameters	2.995	1.734–5.173	< 0.001

## Discussion

In this retrospective study, we aimed to use baseline ^18^F-FDG PET radiomics features and clinical and conventional PET metabolic parameters to develop models for predicting the 2-year PFS and 5-year OS in patients with DLBCL and compare their predictive ability. To the best of our knowledge, this is the first study to compare the prognostic value of these three models. The major finding of the present study was that all three models could be used to facilitate individualized prognostic prediction in patients with DLBCL. Simultaneously, in comparison with clinical factors and PET metabolic parameters, radiomics signatures had the best predictive performance for PFS and OS, with the lowest AIC value and the highest C-index value.

Our results showed that radiomics signatures could stratify high-risk patients with early recurrence and poorer survival using a noninvasive method and then optimize personalized treatment strategies that will benefit these patients. This finding corresponds to the results of a recent study in which several single radiomics features were independent predictors of survival outcomes [16, 30]. However, our radiomics signature differs from previous studies in that it captures a broader range of underlying features, which were calculated by combining optimal radiomics features from 1328 features per image, consisting of 16 shape descriptors and features extracted from original and derived images. Similarly, another study found that radiomics signatures were independently associated with PFS (HR = 4.150) and OS (HR = 4.029), indicating their predictive value [31]. In comparison, the univariate HRs of radiomics signatures in our study were higher for PFS (HR = 8.546) and OS (HR = 8.367), which indicated that our radiomics signatures may improve risk stratification. Our study, along with previous studies, supports the notion that key radiomics features strongly correlate with intratumoral metabolic heterogeneity and that angiogenesis plays a significant role in disease progression and survival in patients with DLBCL [32, 33].

A previous study [34] showed that the NCCN-IPI outperformed the IPI in risk categorization and 5-year OS estimation in patients with DLBCL. Additionally, a meta-analysis demonstrated that the β2-MG level was an independent prognostic factor in patients with NHL [35]. Our study was the first to choose the combination of β2-MG level and NCCN-IPI as a predictor to build a clinical model, and the results showed that the clinical scores derived from these factors were independently associated with both PFS and OS. High clinical scores have been proven to increase the risk of recurrence and mortality in patients with DLBCL. However, in the present study, clinical factors did not contribute to distinct risk stratification as effectively as radiomics features for PFS and OS. This finding is inconsistent with the results of a previous study [18], which demonstrated that radiomic signatures did not show significantly higher AUCs than the IPI for PFS and OS. One potential explanation for this inconsistency is that the clinical variables used in our model may not have captured all the relevant prognostic information, since there may have been other factors influencing the prognosis of patients with DLBCL that were not included in our study.

Our results further support the potential of metabolic parameters as a noninvasive and quantitative approach for DLBCL prognosis prediction. The metabolic parameters scores were independently associated with PFS and OS. This finding is consistent with the results of previous studies that showed the benefit of incorporating TMTV along with parameters such as Dmax or SDmax to enhance risk stratification and inform treatment decisions in patients with DLBCL [13, 36]. Eertink et al.[37] showed that a model combining MTV, SUVpeak, and Dmaxbulk showed the best predictive ability after 2 years. Furthermore, another study [37] showed that the addition of complex textural radiomics features did not provide additional predictive power compared to dissemination features. In contrast to these findings, our study showed that in comparison with the radiomics signatures, metabolic parameters had lower power to discriminate high-risk patients based on the 5-year OS. One possible explanation for this discrepancy is that conventional PET parameters do not consider the heterogeneity and complexity of tumors and do not provide tumor morphological information, which may limit the predictive power of metabolic parameters for DLBCL prognosis.

Our study also demonstrated that the combined model incorporating both radiomics signatures and clinical factors/metabolic parameters showed a slight improvement in model performance over the use of radiomics signatures alone. This was evidenced by a reduction in the AIC value and an increase in the C-index value for both the 2-year PFS and 5-year OS. However, the difference in performance between the combined model and the radiomics signatures alone was not statistically significant for either PFS or OS.

In our study, a semiautomated method using a fixed SUV threshold of 4.0 (SUV ≥ 4.0) was applied for TMTV segmentation. This approach required less manual adaptation and has been recommended and used to evaluate the prognostic performance [21, 38–40]. However, there is no consensus on the standardized segmentation methods for calculating radiomics features in DLBCL, and this remains an active area of research for future investigations [24]. Although we observed differences among different PET/CT scanners (PHILIPS GEMINI GXL16 or PHILIPS Vereos), our statistical analysis did not reveal any significant statistical differences in prognosis between the two scanners in our dataset. Thus, the choice of scanner may not have had a significant impact on prognostic outcomes in our study population.

This study had several limitations that require consideration. First, this was a retrospective single-center study with a relatively small sample size and lacked external validation, which may have overestimated the performance of the models and limited the generalizability of the findings. Second, the molecular genetic subtypes of DLBCL were not included because of incomplete data. In the future, more robust models should be developed and validated for DLBCL risk prediction in larger multicenter studies.

## Conclusions

In conclusion, radiomics signatures demonstrated the high predictive power for progression in terms of 2-year PFS and 5-year OS in patients with DLBCL. Although baseline PET radiomics signatures, clinical factors, and metabolic parameters showed the potential for individualized prediction, the combination of these three factors may not significantly enhance the predictive value beyond that of the radiomics signatures alone, thus simplifying the decision-making process for clinicians. In summary, the radiomics signatures derived from PET images hold promise as an independently prognostic tool for DLBCL, and further research is needed to explore their clinical utility and potential integration with other prognostic factors.

### Supplementary Information

**Additional file 1. Table S1. **Title of data: ^18^F-FDG PET/CT image acquisition parameters.

**Additional file 2. Table S2.** Title of data: The full list of extracted radiomic features.

## Data Availability

The datasets used and/or analyzed during the current study are available from the corresponding author on reasonable request.

## Abbreviations

DLBCL: Diffuse large B-cell lymphoma
NHL: Non-Hodgkin lymphoma
R-CHOP: Rituximab, cyclophosphamide, doxorubicin, vincristine, and prednisone
IPI: International Prognostic Index
LDH: Lactate dehydrogenase
NCCN: National Comprehensive Cancer Network
β2-MG: β2-Microglobulin
^18^F-FDG PET: ^18^F-fluorodeoxyglucose positron emission tomography
TMTV: Total metabolic tumor volume
PFS: Progression-free survival
OS: Overall survival
VOI: Volumes of interest
BSA: Body surface area
LASSO: The least absolute shrinkage and selection operator
ROC: Receiver operating characteristic
AIC: Akaike information criterion
C-index: Harrell’s concordance index
AUC: Area under the curve
HR: Hazard ratio
